# Development and Evaluation of a Low-Cost Phantom Model for Ultrasound-Guided Intercostal Chest Drain Insertion

**DOI:** 10.7759/cureus.109657

**Published:** 2026-05-25

**Authors:** Robert Pritchard, Eren Keskin, Muhammed Shahid, Johann Willers, James Chu, Alexander Hall

**Affiliations:** 1 Simulation, Brighton & Sussex Medical School, Brighton, GBR; 2 Anaesthetics, University Hospitals Sussex NHS Foundation Trust, Brighton, GBR

**Keywords:** chest drain, low-cost, medical education, phantom model, pleural effusion, procedural skills, simulation, ultrasound

## Abstract

Introduction

Intercostal chest drain (ICD) insertion is a key procedure across multiple specialties within the NHS, used primarily to manage pleural effusions and pneumothoraces. Despite its importance, ICD insertion carries a significant complication rate, including injury to surrounding structures, infection, and bleeding. Ultrasound (US) guidance reduces complication and failure rates and is recommended by the British Thoracic Society for pleural procedures. However, many clinicians report low confidence in US-guided ICD insertion, partly due to limited access to training. Existing training models are often costly or limited by realism and ethical concerns. This study aimed to develop and evaluate a low-cost, realistic phantom model for simulation-based training in US-guided ICD insertion.

Methods

The model, SONIC (SONographic Intercostal Chest drain phantom), was developed using inexpensive, readily available materials, with a final cost of less than £50. The model incorporates anatomically and sonographically realistic ribs, intercostal muscles, and internal organs (lung and liver). SONIC was evaluated during two simulation sessions (February and May 2025) led by Emergency Medicine consultants. A total of 17 participants (including 10 trainees) completed an online questionnaire, with trainees completing additional pre- and post-intervention assessments. Outcomes included self-reported confidence, perceived realism and usefulness, and an informal comparison with commercial models. Data were analysed using Wilcoxon signed-rank tests.

Results

Trainees demonstrated improvements in all pre- and post-intervention measures, with significant improvements in understanding of US-guided ICD insertion (*p* = 0.033) and equipment use (*p *= 0.015). All participants considered SONIC a useful training tool, with 92% agreeing that trainees should practise on the model prior to performing the procedure on patients. Most participants reported realistic US (88%) and tactile (77%) experiences. Among those with prior experience of commercial models, 100% rated SONIC as non-inferior.

Conclusions

Findings from this pilot study suggest that SONIC could represent a low-cost, scalable alternative to existing simulation models for US-guided ICD insertion. Despite a small sample size, the results indicate that the model improves trainee confidence and provides a realistic training experience. Wider adoption of such models may enhance access to simulation training and improve procedural safety.

## Introduction

Intercostal chest drain (ICD) insertion is a common procedure performed across NHS hospitals for the investigation and management of conditions such as pleural effusions, pneumothoraces, haemothoraces and empyema [1]. ICDs play a vital role in multiple specialties, including surgery, intensive care, respiratory medicine, and emergency medicine. Despite its importance, ICD insertion carries a complication rate of approximately 19%, including injury to local structures, pneumothorax, bleeding, infection, and drain blockage or displacement [2].

Ultrasound (US)-guided ICD insertion reduces complication and failure rates compared to non-image-guided techniques [3]. In response to concerns regarding patient safety, including findings from a 2008 National Patient Safety Agency (NPSA) report that identified multiple preventable deaths related to ICD insertion, the British Thoracic Society (BTS) guidelines recommend the use of US guidance for all pleural drainage procedures [4,5]. Real-time US guidance, rather than the ‘X-marks-the-spot’ approach, is increasingly preferred for ICD insertion [6]. Studies report success rates of 93-100% when performed by experienced clinicians [7,8].

The NPSA report highlighted factors such as operator inexperience and inadequate training as key contributors to adverse events. Simulation-based training improves both confidence and procedural competence [9,10]. It is also recommended by national guidelines and training curricula [5,11]. Despite these recommendations, training opportunities remain limited and many clinicians continue to report low confidence in performing US-guided ICD insertion [12].

Existing training models are predominantly commercial or animal-based [13]. Commercial models are often expensive (frequently exceeding £3,000) and lack realism [13,14]. Meanwhile, animal models raise concerns regarding deterioration, anatomical differences and ethics [15]. As a result, there is growing interest in low-cost, realistic, and reusable alternatives. This study aimed to develop and evaluate a low-cost, reproducible phantom model for US-guided ICD insertion and assess its effectiveness as a simulation training tool.

## Materials and methods

Model development

**Figure 1:**
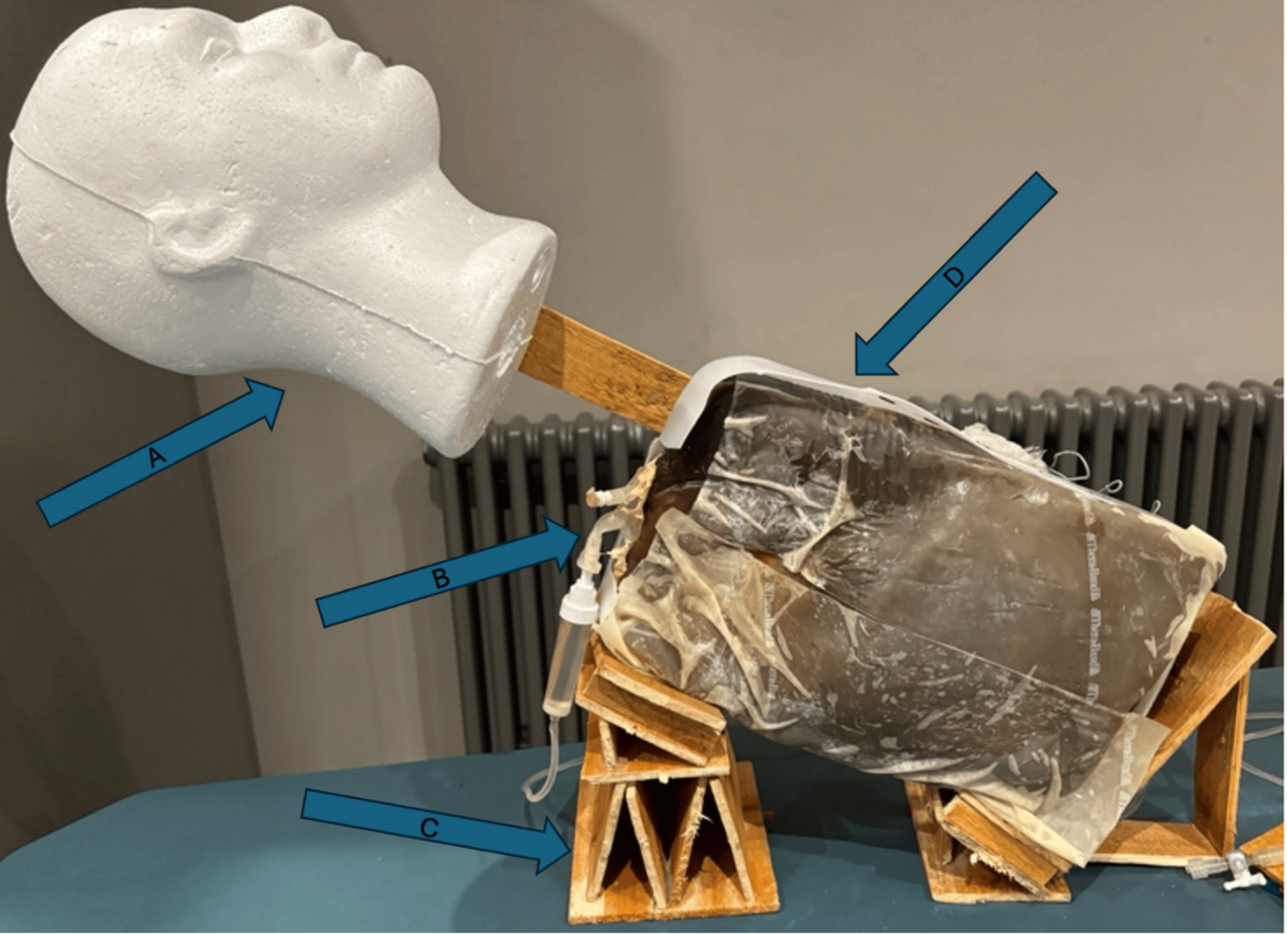
SONIC A) Polystyrene head. B) Giving set used to refill the fluid. C) Wooden stand. D) The main body of SONIC showing the skin (exercise band) and subcutaneous tissue (ADAMgel) underneath the skin. SONIC: SONographic Intercostal Chest drain phantom

The phantom was developed through an iterative process, with successive versions refined based on expert feedback to improve anatomical realism and functionality. The final model, SONIC (SONographic Intercostal Chest drain phantom) (Figure 1), was designed to replicate the key anatomical and sonographic features encountered during US-guided ICD insertion. 

**Figure 2:**
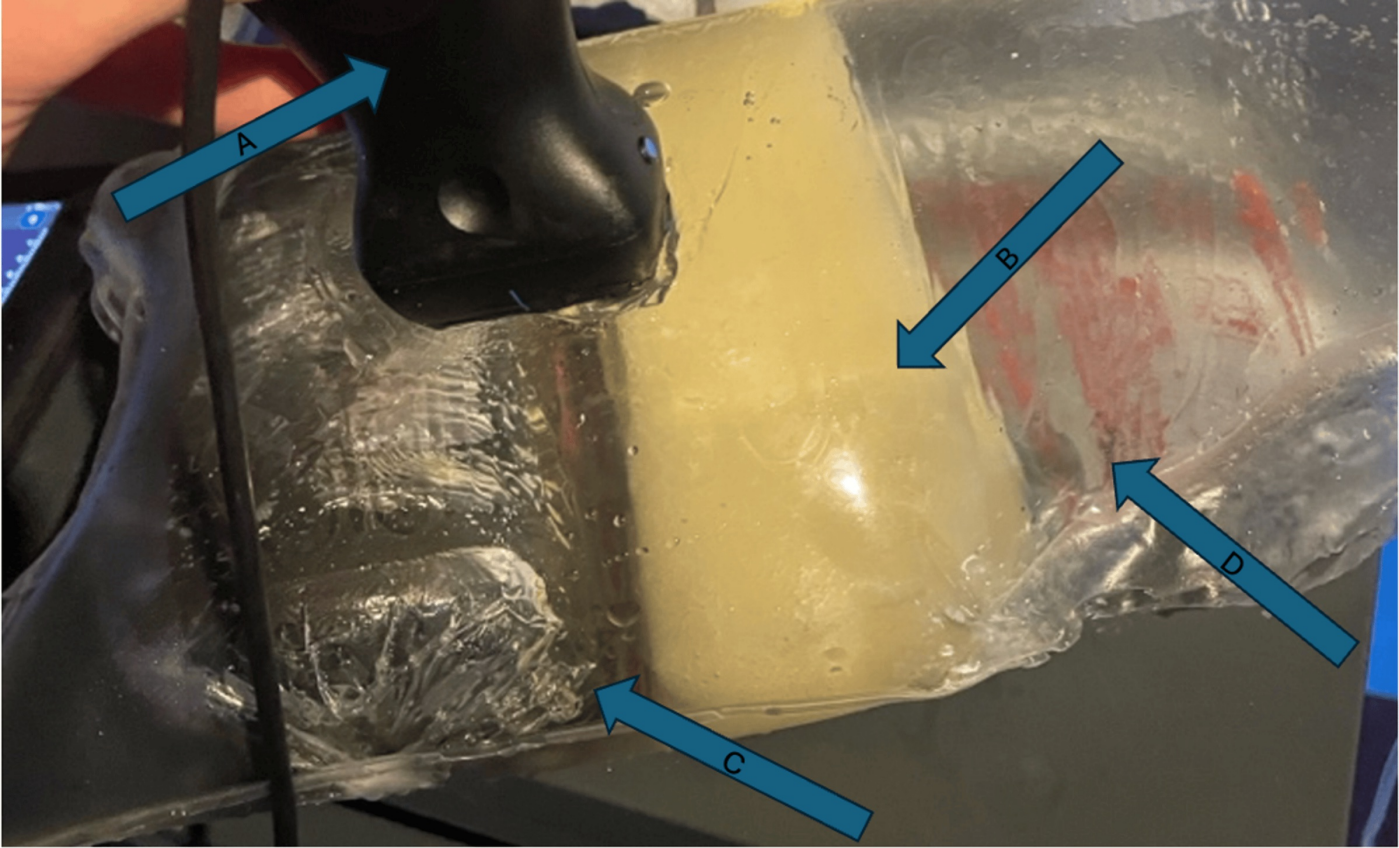
SONIC before adding the latex to the saline bag A) US probe. B) Lung (sponge). C) Liver and diaphragm (ADAMgel wrapped in clingfilm). D) Pleural effusion (sealed fluid-filled 3L saline bag). SONIC: SONographic Intercostal Chest drain phantom

A 3L saline bag was used to simulate a pleural effusion. Internal structures were incorporated by placing a roll of ADAMgel (Aqueous Dietary fibre Antifreeze Mix gel - as described by Willers et al. [16]), tightly wrapped in cling film and sealed to create a watertight structure representing the liver and diaphragm, alongside a sponge to mimic lung tissue. These components were secured within the bag, which was then resealed using heat and adhesive and refilled via a giving set, producing a fluid-filled cavity with identifiable internal structures on US (Figure 2). 

**Figure 3:**
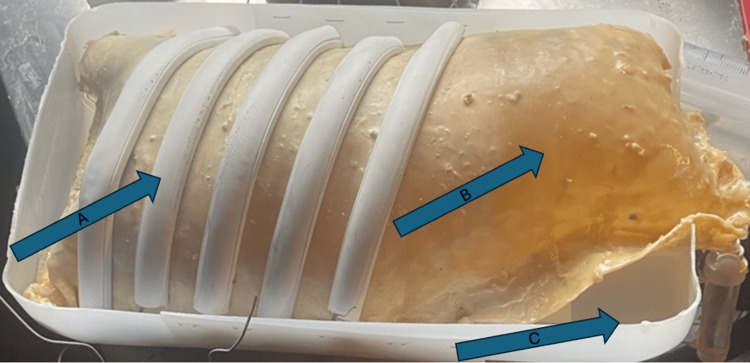
SONIC after adding the latex and cable trunking to the saline bag A) Ribs (cable trunking). B) Parietal pleura (latex). C) Plastic container to support the fluid bag. SONIC: SONographic Intercostal Chest drain phantom

The bag was coated with multiple layers of liquid latex to simulate the pleura and allow repeated needle puncture with self-sealing properties. It was then placed within a plastic container, over which a ribcage was constructed using cable trunking secured with aluminium wire (Figure 3). 

Intercostal muscles were simulated by pouring ADAMgel mixed with chia seeds between the ribs, followed by a layer of ADAMgel to represent subcutaneous tissue. A latex exercise band layer was applied to mimic skin. The completed model was mounted on a wooden stand with an attached polystyrene head, allowing positioning at approximately 30° to replicate patient positioning and facilitate anatomical orientation. The total cost of constructing the phantom was less than £50. Detailed construction steps are available from the authors on request.

Study design and setting

SONIC was evaluated during two point-of-care US simulation sessions held in February and May 2025 at East Sussex Healthcare NHS Trust (ESHT), facilitated by Emergency Medicine consultants. Participants were recruited from attendees at scheduled teaching sessions and received a structured teaching session on US-guided ICD insertion using SONIC. This included identification of a safe insertion site using US, followed by US-guided needle insertion and placement of a Seldinger chest drain (Rocket 18G).

Outcome measures

Participants completed an online questionnaire (see appendix) comprising pre- and post-intervention components. The questionnaire was adapted from a previously published simulation-based assessment tool [17].

To assess the impact of SONIC on trainee confidence, participants rated five statements using a 5-point Likert scale (1/5 = Strongly Disagree, 2/5 = Disagree, 3/5 = Neutral, 4/5 = Agree, 5/5 = Strongly Agree). Participants who selected agree, neutral, disagree, or strongly disagree for any statement were classified as trainees. Those who strongly agreed with all pre-intervention confidence statements were excluded from the trainee group, as they demonstrated maximal baseline confidence.

Additional items evaluated the perceived realism and educational value of SONIC, as well as comparisons with previously used commercial models. Participants were also asked whether they felt there were sufficient training opportunities within their institution for practising US-guided ICD insertion.

Qualitative feedback was collected through free-text responses, allowing participants to describe strengths of the model and suggest areas for improvement.

Statistical analysis

Statistical analysis was performed using IBM SPSS Statistics for Windows, Version 31 (Released 2025; IBM Corp., Armonk, New York, United States). Pre- and post-intervention confidence scores were compared using the Wilcoxon signed-rank test, with statistical significance set at p < 0.05.

Ethical approval

This study was registered with the clinical governance team at ESHT as a quality improvement project. Formal ethical approval by a research ethics committee was therefore not required. Participation was voluntary, informed consent was obtained from all participants, and all questionnaire responses were anonymised.

## Results

Participant demographics

Across the two study dates, 17 clinicians participated, including a Foundation Year 1 (FY1) doctor (n = 1), Senior House Officers (SHOs; n = 7), Registrars (n = 6), and Consultants (n = 3). Of these, 10 were classified as trainees (FY1, n = 1; SHOs, n = 5; Registrars, n = 4). Among trainees, prior experience performing US-guided ICD insertion ranged from 0 to >5 procedures.

Pre- and post-intervention results

Trainees demonstrated improved confidence across all measures. Statistically significant improvements were observed for understanding how to insert a US-guided ICD (p = 0.033) and how to use the required equipment (p = 0.015) (Table 1). 

**Table 1:** Likert scale data for pre-and post-intervention trainee confidence in performing US-guided ICD insertion (n = 10). 1 = strongly disagree, 2 = disagree, 3 = neutral, 4 = agree, 5 = strongly agree. * = statistically significant improvement in before and after scores at *p* < 0.05. ICD: Intercostal chest drain

		Before			After		
Statement	Median	IQR	Range	Median	IQR	Range	*P*-value
I understand the definition of the procedure	4	3.75-5	3-5	4	4-5	3-5	0.48
I understand how to perform the procedure	4	2.75-4	2-5	4	4-5	3-5	0.033*
I understand the relevant anatomy	3.5	3-4.25	3-5	4.5	4-5	3-5	0.053
I understand how to use the equipment required	3.5	2-5	2-5	4.5	4-5	4-5	0.015*
I would be able to perform the procedure	4.5	1-5	1-5	4.5	3.75-5	3-5	0.066

Model evaluation

All 17 participants provided feedback on the realism and training utility of SONIC (Table 2); 76.5% agreed that SONIC resembles the real thing on touch, and 88.2% agreed it resembles the real thing on US. All participants agreed that SONIC would be a useful training aid, and 92.3% (n = 13 due to missing data) agreed that trainees should use SONIC before practising on patients. 

**Table 2:** Participant evaluation of SONIC *Agree = Likert 4-5/5, Strongly agree = 5/5 SONIC: SONographic Intercostal Chest drain phantom

Statement	% Agree (Strongly agree)*
The model resembles the real thing on touch	76.5 (41.2)
The model resembles the real thing on US	88.2 (29.4)
The model would be a useful training aid for trainees	100 (58.8)
Trainees should use this model before practising on patients	92.3 (46.2)

Among participants who had previously used commercial models (n = 11), five participants agreed that SONIC was better and six participants agreed that it was equivalent to previous models.

Qualitative feedback

Eleven participants provided free-text comments, describing SONIC as “very detailed”, “realistic”, “best I’ve seen” and “easy to use”.

Training opportunities

Regarding the statement "There are enough training opportunities within the trust for practising adult US-guided chest drain insertion", four participants strongly disagreed, eight participants disagreed, four participants were neutral, and one participant agreed.

## Discussion

This study aimed to develop an inexpensive, reproducible, and realistic phantom model for practicing US-guided ICD insertion. The results indicate that SONIC successfully achieves this objective, as evidenced by increased trainee confidence following its use.

**Figure 4:**
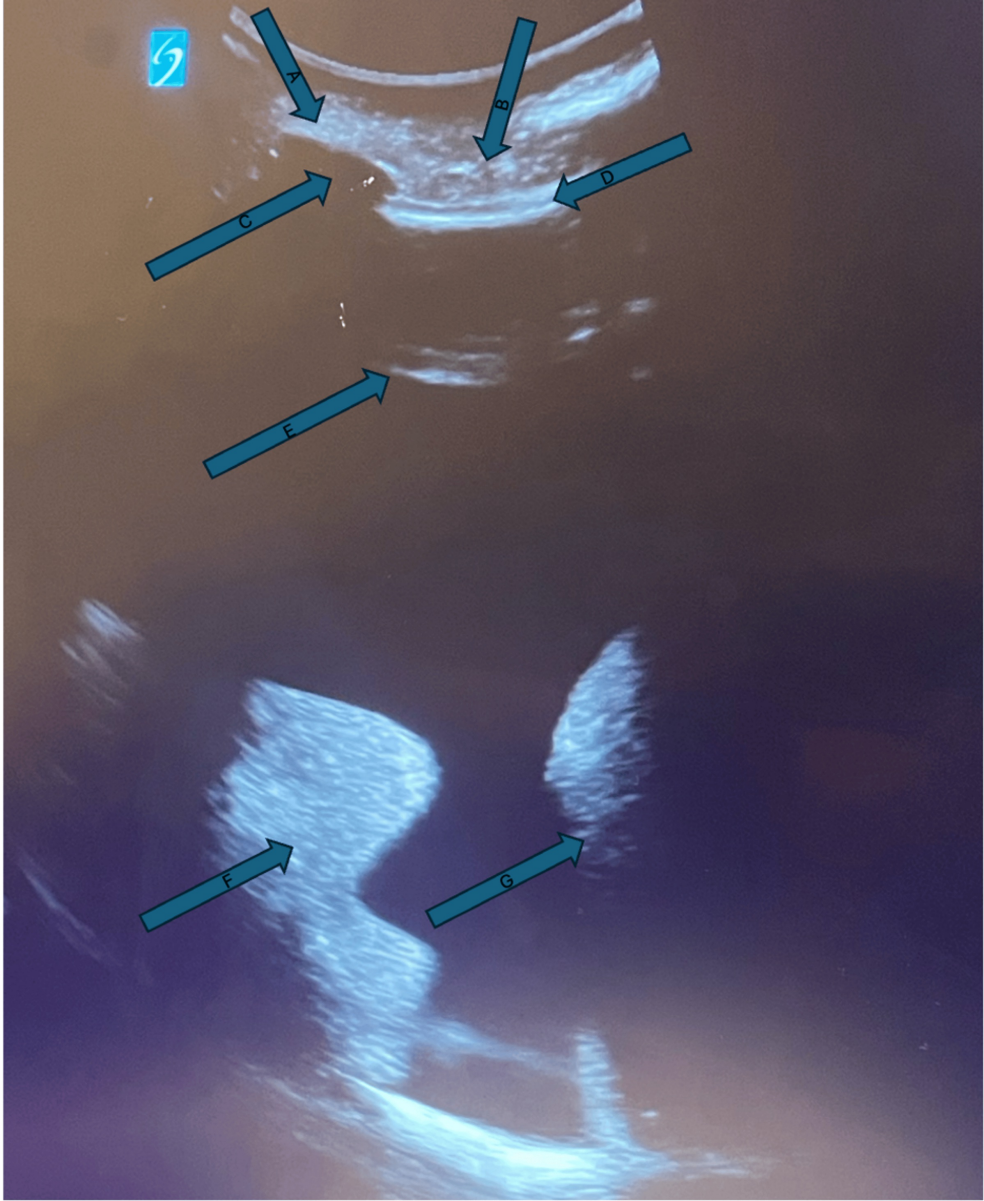
A US image of SONIC A) Subcutaneous tissue. B) Intercostal muscle. C) Rib. D) Parietal pleura. E) Needle tip within the pleural cavity. F) Lung. G) Liver. SONIC: SONographic Intercostal Chest drain phantom

**Figure 5:**
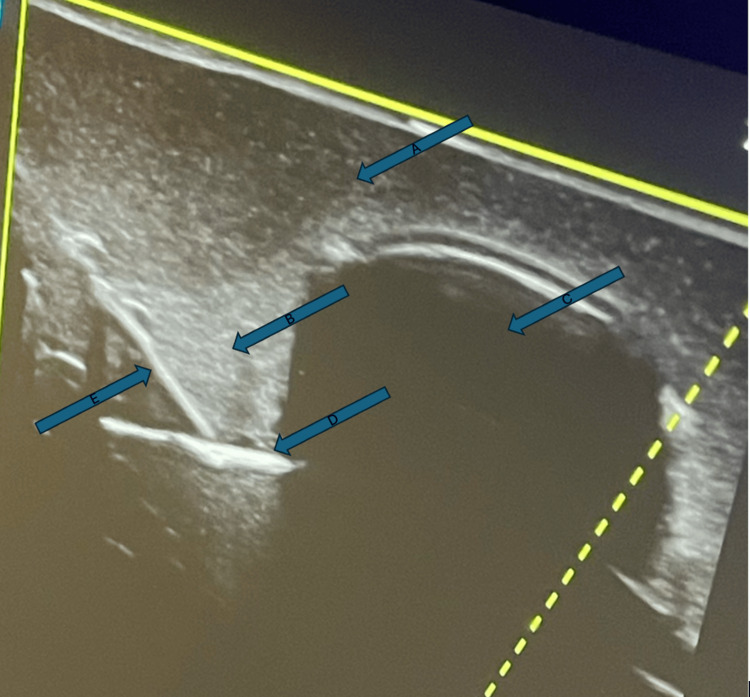
A US image of SONIC showing a needle entering through the parietal pleura A) Subcutaneous tissue. B) Intercostal muscle. C) Rib. D) Parietal pleura. E) Needle entering through the parietal pleura. SONIC: SONographic Intercostal Chest drain phantom

**Figure 6:**
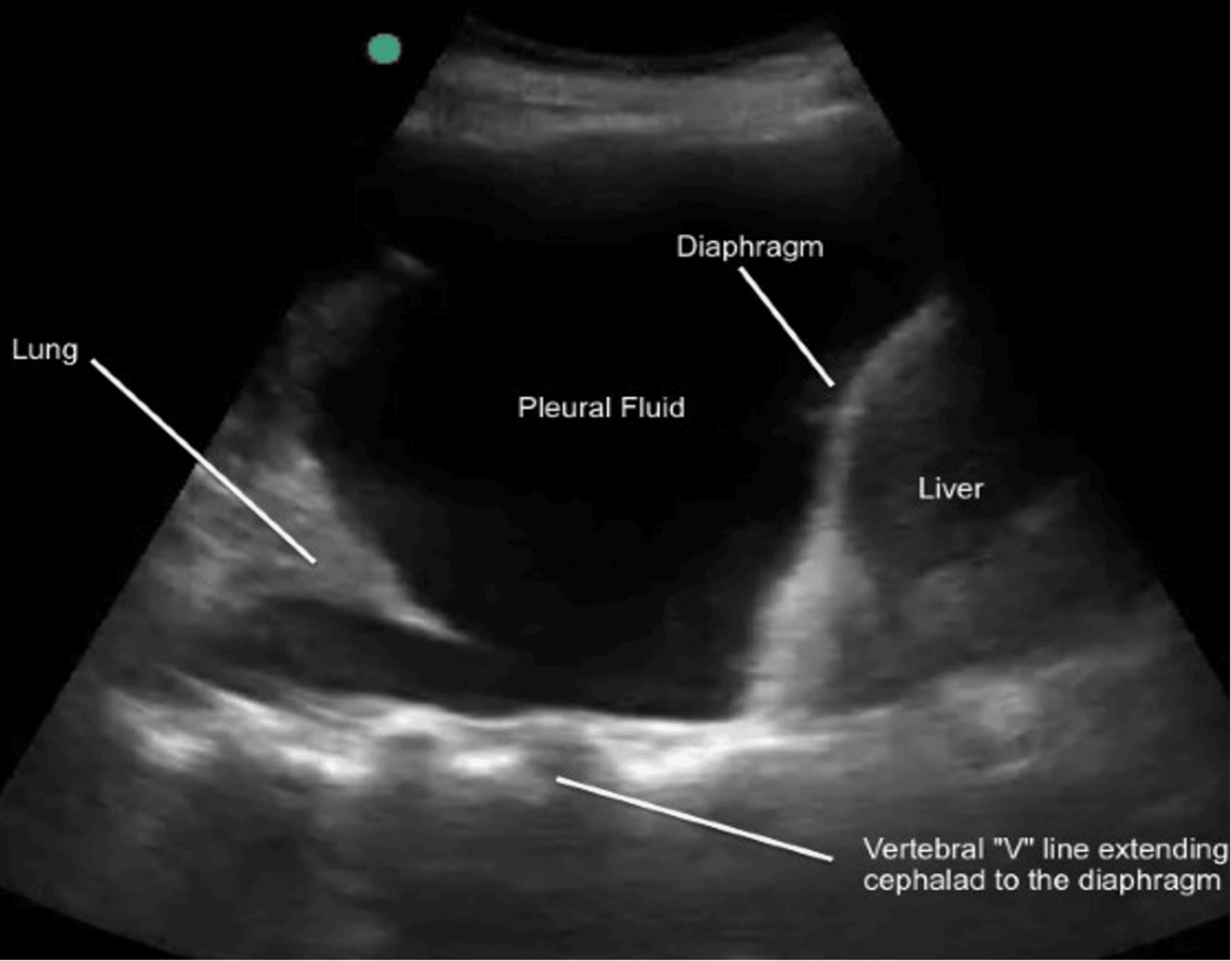
A US image showing a real pleural effusion Reproduced from the study by Atkinson et al. [18] under the terms of the Creative Commons Attribution 2.0 International License.

SONIC meets key criteria for an ideal phantom, including anatomical accuracy and US realism. Unlike many homemade models, SONIC allows needle insertion and visualization within a simulated pleural effusion, replicating relevant structures such as the lung, ribs, intercostal muscles, and liver. The free-text feedback often described SONIC as realistic, particularly when viewed using US (Figures 4, 5). Figure 6 shows a real US image of pleural effusion for comparison with that produced by SONIC [18]. This anatomical fidelity provides a closer approximation to clinical conditions, supporting skill acquisition, as mid- to high-fidelity simulation has been shown to improve procedural competence compared to low-fidelity alternatives [19,20]. SONIC is constructed without animal products, reducing ethical concerns and extending model longevity. 

Participants indicated that SONIC performs comparably to commercial models, although this assessment was based on a single-question, subjective comparison, and direct side-by-side evaluation was not conducted. Future studies could include controlled comparisons to further investigate this finding.

These findings support existing evidence highlighting the value of simulation-based training for pleural procedures [21]. US-guided ICD insertion is complex and carries a high risk of complications, often limiting hands-on opportunities for trainees [22]. This study indicates that access to simulation-based ICD insertion training remains limited within the trust, despite BTS guidelines encouraging simulation-based practice [5]. This mirrors broader trends, as a 2015 survey of higher specialty and core medical trainees in southern England found that nearly 50% had never received simulation training for ICD insertion, and over 60% lacked training in thoracic US [22]. SONIC could help address this gap by offering a low-cost, high-fidelity model that could be implemented more widely. Open-access construction guides could facilitate broader adoption. 

While models like SONIC are valuable training tools, trainees should not rely on task-trainers alone to increase competency [23]. It is recommended that, following simulation, trainees complete five to seven supervised ICD insertions on patients to achieve full competency, followed by five to ten per year to maintain their skills [13,22]. Regular simulation could help reduce the number of clinical insertions required to maintain competency, as even one simulation session per year improves confidence [24]. 

Limitations

This study has several limitations. Although adapted from a validated questionnaire, the questionnaire has not been formally validated, introducing potential bias and limiting generalisability of results. The single-centre design and small sample size (10 trainees) reduce statistical power and further limit the applicability of the findings. Social desirability bias may have influenced responses, as participants could have felt compelled to provide positive feedback. Larger studies, particularly ones comparing SONIC to other models, would be needed to confirm its effectiveness. Similar studies validating models for ICD insertion typically include 30-80 trainees [25-27].

This study assessed self-reported confidence, a subjective measure that may not directly reflect procedural competency or improved patient outcomes, particularly as high-fidelity simulation may lead to overconfidence [28,29]. As effective simulation relies on expert assistance [23], it is difficult to determine whether the observed results were due to SONIC itself or the quality of instruction provided. While SONIC has not yet been used to assess trainee procedural competency, other ICD simulators have demonstrated this potential [10]. Further studies could incorporate SONIC into structured teaching programmes to teach and assess US-guided ICD insertion, evaluating its impact on procedural competency.

SONIC also has a few structural and practical limitations that could be addressed through further development. Currently, it replicates only right-sided pleural effusions, although a left-sided version could be created by adjusting the stand and replacing the liver with a spleen and heart. The model is not suitable for individuals with latex allergies, as both the skin and pleura are made from latex. Constructing SONIC is also time-consuming, taking most of a day. 

Repeated insertion of needles and drains can cause minor leakage, although the model has largely self-sealed once drains are removed. Occasionally, the fluid bag requires refilling, which can be done using a syringe attached to the giving set. No significant maintenance, such as patching holes or replacing ADAMgel tissue, has been required to date, though this may become necessary with prolonged use. 

## Conclusions

This study successfully developed and evaluated SONIC, a novel, low-cost, and easily reproducible phantom for US-guided ICD insertion simulation. The results indicate that SONIC enhances trainee confidence and provides a realistic simulation of pleural effusion drainage. Trainees and experienced clinicians alike highly rated its sonographic appearance and tactile realism, distinguishing it from existing training models.

While the small sample size and reliance on subjective measures limit the strength and generalisability of findings, this pilot study provides proof-of-concept for the use and scalability of SONIC in medical training. Further research should focus on refining its design and conducting larger studies to validate its impact on procedural competence. By providing an effective alternative to commercial and animal-based models, SONIC could play a crucial role in improving US-guided ICD insertion simulation and patient safety across healthcare settings.

## Appendices

**Table 3:** Microsoft Forms questionnaire and participant responses

ID	Grade	Specialty	Previous experience of adult US-guided chest drains	How much do you agree with the following statement: There are enough training opportunities within the trust for practising adult US-guided chest drain insertion	BEFORE: I understand the definition of inserting an US-guided chest drain	BEFORE: I understand how to insert an US-guided chest drain	BEFORE: I understand the anatomy relevant to inserting an US-guided chest drain	BEFORE: I understand how to use the equipment required in an US-guided chest drain	BEFORE: I would be able to perform an US-guided chest drain on an adult patient	AFTER: I understand the definition of inserting an US-guided chest drain	AFTER: I understand how to insert an US-guided chest drain	AFTER: I understand the anatomy relevant to inserting an US-guided chest drain	AFTER: I understand how to use the equipment required in an US-guided chest drain	AFTER: I would be able to perform an US-guided chest drain on an adult patient	The model resembles the real thing on touch	The model resembles the real thing on ultrasound	The model would be a useful training aid for trainees	Trainees should use this model before practising on patients	IF you have used previous COMMERCIAL models for adult US-guided chest drains, how does this model compare?	Please give any free-text feedback on the model
1	SpR	Emergency medicine	Done once on a patient	Neutral	4	4	5	5	5	5	5	5	5	5	4	4	4		Equal	Liked the thought process and the ribs and intercoastal space feel realistic, also able to identify the fluid in the pleural space.
2	FY1	Respiratory medicine	Watched a few	Disagree	3	2	3	2	1	4	4	5	4	3	5	5	5		Better	The texture and ultrasound ability was really good
3	Middle grade (IMT, ST1-3 etc)	IMT	Done 5+ times on patients	Strongly disagree	5	4	3	4	5	5	5	5	5	5	5	4	4	4	Better	Very detailed
4	SpR	Emergency medicine	Done 5+ times on patients	Disagree	5	4	4	4	5	4	4	4	5	5	4	4	5	5		
5	Middle grade (IMT, ST1-3 etc)	Anaesthetics/ intensive care	Done once on a patient	Disagree	4	2	3	2	1	4	4	4	4	3	5	4	5	4		Felt realistic, particularly useful as this is a difficult procedure to come across
6	FY2+	Emergency medicine	Watched a few	Neutral	5	3	3	3	3	4	4	4	4	4	4	4	4	4	Equal	
7	FY2+	Acute medicine	Done once on a patient	Disagree	4	4	4	3	4	3	3	3	4	4	2	2	4	2		
8	Middle grade (IMT, ST1-3 etc)	Acute medicine	Been shown how to do it but not done on a patient	Strongly disagree	3	3	3	2	1	4	4	4	4	4	3	3	4	4		
9	SpR	Emergency medicine	Done 2-4x on patients	Agree	4	4	4	5	5	5	5	5	5	5	4	4	4	4	Equal	
10	SpR	Emergency medicine	Done 5+ times on patients	Neutral	4	5	5	5	5	5	5	5	5	5	4	4	5	5	Better	Better than once I have practiced on before
11	Consultant	Emergency medicine	I teach people how to do them	Strongly disagree	5	5	5	5	5	5	5	5	5	5	4	4	5	5	Equal	Easy to use. Love the built in head
12	Consultant	Emergency medicine	Nothing	Disagree	5	5	5	5	5	5	5	5	5	5	3	5	5	5		Brilliant, very much life like images
13	SpR	Radiology	Done 5+ times on patients	Disagree	5	5	5	5	5	5	5	5	5	5	5	5	5		Better	Good realistic models which can help radiology build on their ultrasound guided procedural skills.
14	SpR	Respiratory medicine	Done 5+ times on patients	Neutral	5	5	5	5	5	5	5	5	5	5	3	4	4		Equal	Sustainable, resemblance with other models I have used before, the ability to use the USS on this model to define rib spaces, ribs, pleura and fluid, liver edge
15	FY2+	Still yet to commit (e.g. foundation)	Nothing	Disagree	5	5	5	5	5	5	5	5	5	5	5	5	5	5	Equal	
16	FY2+	Emergency medicine	Watched a few	Disagree	5	5	5	5	5	5	5	5	5	5	5	5	5	5		Very knowledgeable and kind instructor. Helpful model.
17	Consultant	Emergency medicine	I teach people how to do them	Strongly disagree	5	5	5	5	5	5	5	5	5	5	5	4	5	4	Better	best I've seen
